# Applying a scoping review approach for identifying effective implementation strategies in oral health settings

**DOI:** 10.1017/cts.2021.857

**Published:** 2021-09-20

**Authors:** Erick G. Guerrero, Charles D. Kaplan, Inga Gruß, Julie Frantsve-Hawley, Jeffrey L. Fellows, Nadia Yosuf, Deborah E. Polk

**Affiliations:** 1 I-Lead Institute, Research to End Healthcare Disparities Corp, Los Angeles, CA, USA; 2 Neurology Department, University of Southern California, Los Angeles, CA, USA; 3 Kaiser Permanente Center for Health Research, Portland, OR, USA; 4 Julie Frantsve-Hawley Consulting, CareQuest Institute for Oral Health, Boston, MA, USA; 5 University of Pittsburgh School of Dental Medicine, Pittsburgh, PA, USA

**Keywords:** Implementation strategies, scoping review, evidence-based practices, rapid methods

## Abstract

Dental service providers have limited capacity to identify strategies to implement evidence-based practices (EBPs). We developed a rigorous yet parsimonious scoping review approach to identify, select, and rate implementation strategies based on an oral health system context. From 153 strategies identified, we selected the top 11 strategies, which had a moderate level of support of evidence and where managers were the main actors. The main actions were to educate, remind, structure, and influence. Targets included dentists, dental hygienists, and assistants and managers from a large prepaid dental care delivery system. This approach responds to calls for rapid and innovative methods to implement EBPs in oral health.

## Introduction

Dental care organizations, like other healthcare organizations, are expected to deliver effective care based on evidence-based practices (EBPs) [1]. However, there are often many barriers that challenge the clinical implementation of EBPs. Generally, there is a lack of a process to ensure EBPs are effectively implemented [2]. Implementation strategies, which are the specific arrangements, facilitators, or conditions supporting implementation, are essential tools for healthcare providers to uptake EBPs [3]. Despite a large number of implementation strategies in use, there is little guidance for healthcare systems to select the strategy that best fits their implementation context [4]. Offering healthcare systems an easy to follow, systematic approach to identify, select, and test implementation strategies may improve efforts to deliver EBPs [5].

There are more than 100 implementation strategies that differ in terms of who initiates implementation efforts, what action is considered in the implementation, who is targeted to carry on the implementation effort, and at what level of influence implementation is conceptualized [3,6,7]. Emerging approaches address these issues of selecting and tailoring implementation strategies [6]. Concept mapping, for example, is a mixed methods approach used for selecting implementation strategies that can help identify and prioritize factors that may affect the implementation process [7]. Another approach is conjoint analysis, which quantitatively identifies implementation strategy profiles to indicate implementer preferences [7]. A third approach is intervention mapping, which enables implementers to gain a better understanding of the existing evidence supporting different pathways of the implementation of specific EBPs or clinical interventions [7]. One shortcoming of all these approaches is that they are generally designed, led, and deployed by researchers outside the healthcare delivery system rather than by members who actually work within healthcare systems.

To identify and rate implementation strategies clearly and directly, the process of helping healthcare systems classify strategies should be based on foundational definitions that consider several dimensions, such as the **actors**, **action, and target** [8] and level. Each of these elements is described in Table 1.

**Table 1.** Description of the model of rating based on actor, action, level, target, and evidence

Focus	Description
Actor	Identifying the people who initiate the action of an intervention can help the selection of implementation strategies because the actor has to be available and willing [8] (e.g., the expert in the train the trainer intervention). Often, the actor introducing or promoting an implementation strategy or intervention is a middle manager [20]
Action	Identifying the action in implementation strategies helps the selection process because it highlights the process of change (e.g. the act of training/teaching in the train the trainer intervention), thereby helping to clarify the mechanism that accounts for efficacy. Researchers and practitioners are increasingly interested in determining the action and the level at which this action is taken (e.g., system, organizational, individual) [21,22]
Level	Describing the level of the strategy helps change agents understand the area of change (e.g. levels at policy, organizational behavior, individual attitudes) [8]. Articulating the level of analysis also helps change agents understand unit and levels of analysis, tailor approaches, and consider resources needed
Target	The target of the strategy is the beliefs or behavior that will change because of the implementation strategy [23]. An implementation strategy or intervention generally seeks to activate employees to alter their pattern of behavior and adopt a new behavior, which represent the clinical intervention or evidence-based practices

It is critical for health care providers to identify strategies that have been shown through empirical research to be effective along an established continuum of evidence (ranging from anecdotal or small evidence to causal linkage or big evidence) [9]. Simultaneously identifying implementation interventions and their respective levels of evidence narrows down the number of implementation interventions to choose from. It also provides an accepted standard of quality of the clinical EBPs to be implemented, all before an investment has to be made to implement the EBP in the field.

Below, we describe a scoping review methodology informed by the Cochran review approach to help healthcare systems in general and dental health systems to identify and classify various evidence-based implementation strategies for stakeholder vetting. Key to the approach is special attention to differences in actors (managers, clinicians, others) and actions (supervising, coaching, guiding) that currently function in the specific system under study. This focus on differentiating the role of actors and actions allows implementers to identify strategies that are most relevant to both the system and to its varying clinical settings. This pragmatic process of scoping implementation strategies puts primary consideration on the lived experience and context of those (providers) who will be most affected by any policy and practice changes. These contextual factors may include external policies and regulation, internal setting conditions, such as workforce issues, training needs, leadership effectiveness, and other factors that may facilitate the implementation of EBPs [10].

## Methods

### Setting and Design

This scoping review is part of the Dissemination and Implementation of Sealant Guidelines in Organizations (DISGO) study funded by the NIH (U01DE027452). The DISGO study is a cluster-randomized, stepped-wedge clinical trial of a deliberative loop (DL) implementation intervention for the purpose of improving the uptake of a evidence-based practice guideline on dental sealants at the Kaiser Permanente Northwest (KPNW) Dental program [11]. KPNW is characterized by several key inner and outer contextual factors that we thought would influence the implementation of the intervention. KPNW is a large integrated health system that provides prepaid medical and dental care to enrolled members. The KP Dental plan (KPD) provides comprehensive care to over 270,000 patients at 21 dental offices in Northwest Oregon and Southwest Washington. Permanente Dental Associates (PDA), a large group practice of general dentists and specialists, provides prepaid dental services to KPD enrollees. The regulation and leadership style of KPD were contextual factors that were also considered. Our study aimed to identify strategies that could be introduced in the DL implementation intervention that are responsive to Kaiser’s staff needs and sensitive to the characteristic contextual factors of the KPNW dental care system. The questions addressed by the scoping review include the role of actor, actions, and level of actions to improve the uptake of EBPs in dental clinics. We relied on the Cochran review methodology [12] and systematic reviews of implementation strategies in oral health [13] to inform the core steps of our scoping review.

### Identifying Implementation Strategies in Peer-Reviewed Publications

In the first stage, we, (IG, CK, EG) identified the peer-reviewed publications with evidence for specific implementation strategies in healthcare settings. We searched five primary electronic databases that included Google Scholar, PubMed, PsycINFO, Cochrane Library, and EBSCO. Our literature searches included the following keywords: implementation strategies, implementation approaches, and implementation facilitators. The inclusion criteria were informed by guidelines used in systematic reviews in implementation science [4,6] and included implementation strategies, EBPs, healthcare, in English language, years 2000–2019. Reviewers were calibrated to the selected criteria. Two rounds of screening were conducted based on abstracts in the first round and full text in the second round. The identified citations were split across the three reviewers, with random audits to verify fidelity to the criteria.

### Systematically Selecting Implementation Strategies Relevant to KPD

At the second stage, we (IG, CK, EG) sorted all implementation strategies into the following categories: dissemination strategies, implementation process strategies, capacity-building strategies, and scale-up strategies using the analytical framework of the role of actor, actions, and level of actions [8]. While sorting strategies into these categories, we removed duplicate strategies. We also removed strategies that were not relevant to the study focus area (e.g. influence policymaking, encourage patients to demand a treatment) or not feasible in the context of KPD (e.g. due to resource constraints). Strategies were screened via rater’s group discussion. We relied on the expert opinion of IG’s organizational expertise at KPD (e.g., leadership and managerial style, organizational norms, and practices) and the expertise in implementation from EG (outer and inner context factors, particularly regulation and leadership style) and clinical interventions (EBPs acceptability and readiness). In this stage, we were guided in the sorting by our main research question of what strategies for implementing the DD would be most relevant to improving the KPD system’s uptake of the EBP of placing dental sealants?

### Scoring

In this third stage, the three raters scored the relevant implementation strategies based on level of evidence. To do so, we conducted an additional targeted literature search to determine the level of available evidence that supports each identified strategy. We adopted a scoring criterion based on Rosseau’s [9] notion of small evidence (grey literature, qualitative) and big evidence (experimental studies leading to metanalysis), whereby 1 represents small evidence and 5 represents big evidence. Raters assigned scores independently and in duplicate, discussed discrepancies in ratings, and reached consensus on final ratings. All strategies rated 2 and lower were removed. The remaining strategies were reviewed by KPD’s quality and operations leadership to assess implementation feasibility, including alignment with organizational vision, timing with competing priorities, and potential resource needs.

### Classifying Implementation Strategies by Actor, Action, Level, and Target

The final stage consisted of classifying strategies independently and in duplicate based on Proctor *et al.*, [8] framework of actor, action, [level], and target as described in the Introduction. The main criteria that guided the classification were clarity in conceptualization and distinctness of the strategy from other strategies. Raters discussed the strategies based on the criteria and reached a consensus on the best practices. The final list of strategies is presented in the following narrative.

## Results

**Fig. 1.**
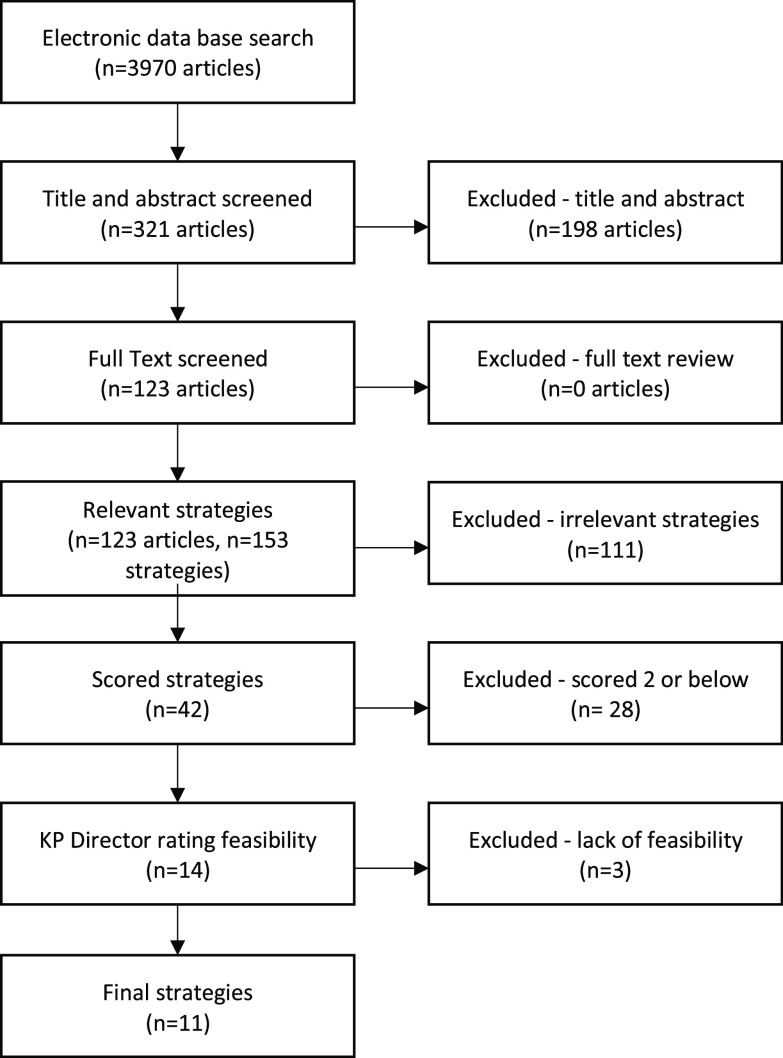
Process of the scoping articles. KP, Kaiser Permanente.

The literature search resulted in 3970 articles. After screening, 123 articles met the inclusion criteria and yielded 153 strategies. Many studies reported more than one strategy. We did not report implementation interventions that were not used for uptake purposes. Figure 1 provides a schematic representation of the identification process. After removing duplicate and irrelevant strategies, 42 strategies remained. After scoring the strategies based on level of evidence, 28 with a rating of low evidence (score of 1–2) were removed, leaving 14 implementation strategies with an evidence score of 3–5. Three additional strategies were removed based on their low feasibility to be implemented in dental clinics. Eleven final strategies resulted from this review. These strategies considered the expert opinion of KPD’s Associate Director based upon the criteria for implementation and feasibility context (e.g., hectic, fast pace, and task-driven work setting) to adopt, follow, and adhere to EBPs such as the intervention considered in the parent study, i.e. dental sealant guidelines.

The 11 strategies (e.g., audit and provide feedback, creating learning environment, assign, and deploy experts) (Table 2) had in common that they all relied on full organization operation, interaction, and technology and human resources. For instance, Audit and provide feedback, as a system strategy has three main actions, referred sometimes in the literature as steps: (1) formulate specific goals and provide agreed-upon feedback; (2) provide protected time for implementation; and (3) evaluate results [14]. The strategy of creating a learning collaborative includes nine steps: (1) develop a framework for online learning via chatrooms; (2) develop listservs; (3) use text messages; (4) conduct educational meetings; (5) conduct outreach visits; (6) deliver training in innovation; (7) develop and distribute educational material; and (8) activate academic partnerships (i.e., Ebert *et al.* [15]). Finally, assign/train and deploy sealant expert in each clinic relies on four steps: (1) identify early adopters identified by colleagues; (2) assign/train NCCL champion for each clinic; (3) provide clinical supervision; and (4) use the train-the-trainer. Our categorization of actors, action, level, and target revealed the following mutually inclusive categories:

- Actor: six strategies focused on leadership, two on supervisors or managers, and five on staff.
- Action: four focused on education, three on reminders, five on structure, and four on influence relevant to oral health.
- Level: seven strategies focused on unit, two in organization, one in management, and one in clinic.
- Target: five focused on all staff, three in dentist staff, and three in leadership/management.

**Table 2.** Actor, action, level, target, and evidence for identified implementation strategies

Implementation strategies (*N* = 11)	Actor	Action	Level	Target	Evidence-base
Audit and provide feedback	Management supervisor	Formulate specific goals and provide agreed upon feedback Provide protected time for implementation Evaluate results	Unit	All staff	3
Create a learning collaborative	Management/clinic staff	Develop a framework for online learning via chatrooms, listserv, text, and more with relevant info on the challenges of NCCL Conduct educational meetings, outreach visits, training in innovation Develop and distribute educational material, activate academic partnerships	Unit	All staff	3
Conduct cyclical small tests of change	Leading clinic staff	Integrate NCCL improvement focus into projects	Unit	All staff	3
Change recording systems to facilitate relay of real-time clinical data	Clinic staff	Step 1: Identify and agree upon best way to use current system to id Step 2: Document and follow up with occlusal lesions Step 3: Use data warehousing techniques to integrate clinical records across systems	Unit	Dentist/staff	3
Assign/train and deploy NCCL expert in each clinic	Staff/leaders	Step 1: Identify early adopters identified by colleagues Step 2: Assign/train NCCL champion for each clinic Step 3: Provide clinical supervision Step 4: Use the train-the-trainer	Clinic	Staff/doers	5
Involve executive boards	Executive leadership	Executive board completes a check list review of implemented changes in clinics, including NCCL	Management	Director/supervisors	3
Provide local and centralized technical assistance	Management/IT staff/technicians	Integrate practice facilitations Technical expert available on the floor to guide practices in time	Unit	Staff	4
Remind clinicians	IT staff	Use data experts Introduce notes in the electronic health record when clinicians document occlusal lesions	Unit	Dentist	3
Model and simulate change	Executive leadership/IT staff	Simulate change that will be implemented prior to sealant introduction	Unit	Change teams of management, dentists and staff	4
Obtain formal written commitments	Executive leadership/management	Encourage to include NCCL sealants in the formal communication of written TPM goals	Organization	Dentists/staff	4
Promote network weaving	Executive leadership/management	Influence KPD staff through positive connections with respected peers and or leaders in the field	Organization	Management/dentists/staff	3

IT: information technology; KPD: Kaiser Permanente Dental; NCCL: non-cavitated carious lesions; TPM, Total Productive Maintenance.

## Discussion

This study presented a description of a pragmatic approach to scoping implementation strategies that are contextually relevant to local dental settings. We relied on a process of identifying, selecting, scoring, and classifying implementation strategies for uptaking EBPs in a real-world health care system. Similar methodological approaches have been developed in other areas of health care [4], but our approach considered that health care practitioners have the most knowledge of whether a given implementation strategy would be feasible and acceptable.

Our approach highlights the parsimony and transparency needed in the field to improve the rigor in the clinical decision-making of health care providers at all levels. Consistent with other reviews in dental practice [11], selected implementation strategies focused on education, reminders, and multifaceted implementation that would apply to the DD intervention. These 11 strategies will be provided in the next stage to KPD practitioners as options to consider in implementing an EBP (e.g., sealant guideline). This approach can inform research designs that highlight the importance of context to improve the implementation process.

Health care providers can determine which among these 11 strategies to select, test, and implement. These systems generally consider a series of factors before committing to implementing a new practice. A few of these factors include cost of the implementation efforts [16], likelihood of implementation success, utility of the implemented practice, and coherence and fit of the new practice with the culture and/or climate of the organization [17].

Our results add a level of rigor to clinical decision-making by reducing the otherwise ambiguous and resource-intensive process of identifying and selecting implementation strategies that can help health care systems effectively deliver EBPs. Engaging stakeholders in the initial steps of implementation can directly contribute to validating their views to improve implementation strategies and delivery of new practices in a real-world health care context.

## Limitations

The proposed approach is limited based on its selection criteria, available literature review, and reach. We limited our search to English-language articles. The scoping review focused on health care systems in the USA and was conducted from research team members also involved in the parent study. This could be a source of unconscious selective bias. Our scoping review is limited in its reliance on existing implementation strategies published in the literature. It does not allow for new strategies that may be suited for the context to be developed anew. Another limitation is that there may be other categorization schemes that may be consistent with the context. We also did not directly evaluate the effectiveness of the proposed implementation strategies but instead relied on the level of evidence in the literature as an indicator of effectiveness. Despite these limitations, the approach presented in this study offers practitioners with a rigorous yet parsimonious way to improve decision-making about what strategies to employ in the implementation of EBPs.

## Conclusion

Our pragmatic approach to scoping implementation strategies provides managers and implementers in dental health care organizations with a pragmatic methodology to select implementation strategies that are context-dependent and supported by an acceptable degree of evidence. This can limit the degree of arbitrary decision-making in selecting strategies in real-world healthcare settings, a critical concern [18–20]. Our approach can deliver a useful tool for bringing order to the chaos of conflicting implementation strategies and give the health care professionals who will be affected the most by changes in policy a greater say in the decisions that affect the trajectories of their respective dental organizations. Using our methodology can inform policy that empowers dental organizations to conduct their own scoping reviews.

## Previous presentations

This work was presented at the 13th Annual Conference on Dissemination and Implementation Science in December 2020, where it was rated as the top poster in its division.
